# Fractionated Radiation Exposure of Rat Spinal Cords Leads to Latent Neuro-Inflammation in Brain, Cognitive Deficits, and Alterations in Apurinic Endonuclease 1

**DOI:** 10.1371/journal.pone.0133016

**Published:** 2015-07-24

**Authors:** M. A. Suresh Kumar, Michael Peluso, Pankaj Chaudhary, Jasbeer Dhawan, Afshin Beheshti, Krishnan Manickam, Upasna Thapar, Louis Pena, Mohan Natarajan, Lynn Hlatky, Bruce Demple, Mamta Naidu

**Affiliations:** 1 Center for Radiological Research, Columbia University, New York, New York, United States of America; 2 GeneSys Research Institute/ Center for Cancer Systems Biology at Tufts University School of Medicine, Boston, Massachusetts, United States of America; 3 Centre for Cancer Research and Cell Biology, Queens University, Belfast, United Kingdom; 4 Department of Psychology, Stony Brook University, Stony Brook, New York, United States of America; 5 Department of Pathology, UTHSCSA, San Antonio, Texas, United States of America; 6 Department of Pharmacological Sciences, Stony Brook University, Stony Brook, New York, United States of America; 7 Biosciences Department, Brookhaven National Laboratory, Upton, New York, United States of America; Universidade do Extremo Sul Catarinense, BRAZIL

## Abstract

Ionizing radiation causes degeneration of myelin, the insulating sheaths of neuronal axons, leading to neurological impairment. As radiation research on the central nervous system has predominantly focused on neurons, with few studies addressing the role of glial cells, we have focused our present research on identifying the latent effects of single/ fractionated -low dose of low/ high energy radiation on the role of base excision repair protein Apurinic Endonuclease-1, in the rat spinal cords oligodendrocyte progenitor cells’ differentiation. Apurinic endonuclease-1 is predominantly upregulated in response to oxidative stress by low- energy radiation, and previous studies show significant induction of Apurinic Endonuclease-1 in neurons and astrocytes. Our studies show for the first time, that fractionation of protons cause latent damage to spinal cord architecture while fractionation of HZE (^28^Si) induce increase in APE1 with single dose, which then decreased with fractionation. The oligodendrocyte progenitor cells differentiation was skewed with increase in immature oligodendrocytes and astrocytes, which likely cause the observed decrease in white matter, increased neuro-inflammation, together leading to the observed significant cognitive defects.

## Introduction

Radiotherapy not only causes acute tissue injury at high doses (>20 Gy of low linear energy transfer (LET) X-rays [1]or protons [2]and about 5–10 Gy of carbon ion [3]), but also results in latent physical damage [4] and variable degree of cognitive impairments, at relatively lower doses [5, 6]. There is strong evidence that ionizing radiation (IR) induced oxidative stress and neuro-inflammation result in diminished neurogenesis and cognitive deficits[7]. Subventricular zone region of hippocampus is the predominant site of neurogenesis, due to presence of precursor cells that migrate and differentiate into neurons and glia[8]. Recent work by Giedenski *et al*.[9] show that oxidative stress *in vivo* seems to be localized to regions of hippocampus enriched in multipotent precursors.

As neurons are post mitotic, studies of glial-restricted precursors in the glial compartment of CNS becomes important, as these cells terminally differentiate into astrocytes, oligodendrocytes (OL), and other glia-restricted precursors such as Oligodendrocyte type 2 astrocytes (O2A). Terminally differentiated OL are critical because their loss leads to demyelination, which impairs neurological function[10]. OL have a slow turnover, and are replenished by oligodendrocyte progenitor cells (OPC). Although mature OL exhibit higher tolerance to low-LET radiation, the high charge, high energy (HZE) radiation studies show that OL precursors were sensitive to lower doses of HZE [11]. Glial-cell damage in response to radiation predominantly involves failure of the OPC to replace OL[12].

Whole body irradiation limits one to acute experiments, so another model was necessary to study latent chronic effects. Rats represent one of most suitable *in vivo* models to study IR induced latent effects on OPC (modeling latent effects in radiotherapy patients or astronauts), as these OPCs are predominantly detected in their spinal cords [13]. High LET carbon beam exposure results in reduction of latency of histopathological/ functional changes in rat spinal cord (as compared to X-rays), so the study of latent effects of protons and particle radiation in spinal cords OPC/ mature OL gains relevance. Spinal cord compression is most observed in thoracic region of most radiotherapy patients[14], so we focused our spinal cord irradiations to thoracic sections, to determine whether 250 MeV protons and 300–600 MeV/n HZE (Si / Fe) ions affect the OPC differentiation, in this region over time.

IR induced oxidative stress generated as reactive oxygen species (ROS) cause DNA damage. To date, no studies have determined the outcome of oxidative DNA damage/ processing in the OPC. The predominant DNA repair used during oxidative DNA damage is base excision repair (BER) and some of the BER enzymes studied in OL such as human MutT homolog and human 8-oxaguanine DNA glycosylase1 have shown significant expression in these cells[15]. DNA-polymerases α, δ and ε (in addition to the DNA polymerases β) were found to be elevated in the oligodendroglia and astroglia from rat brains, possibly owing to increased proliferation, and DNA polymerase β was also expressed in the highest amounts in neurons relative to the glial cells[16].

Research on the key BER protein Apurinic Endonuclease-1 (APE1) has focused on astrocytes and neurons, with no reports in OPC[17, 18]. Partial suppression of APE1 results in lower double-strand breaks (DSB) after exposure to radiation and other studies also indicate a role for BER in DNA strand break formation [19–22]. Thus, it is important to determine whether the low/ high LET radiation induced alteration in APE1 could result in formation of more DSB leading to higher non homologous end joining (NHEJ) [23] or homologous recombination (HR), as suggested by Paap *et al*.[24]. Chronic exposure to radiation induces expression of APE1 and Ku70 (a key player in NHEJ) in mouse cutaneous tissue, resulting in the suppression of HR[25]. APE1 was recently implicated in erythroid bodies differentiation, due to its redox-effector (Ref1) function [26], indicating it may have a role in precursor cell differentiation.

APE1 was reduced in Aluminium induced neuro-inflammation in rat brains [27], but could induce the release of inflammatory cytokines like TNF-∝, IL-6 in macrophages [28] and was elevated in ulcerative colitis[29]; hence we need to determine a correlation, if any between altered APE1 and neuro-inflammation in rat brain/ spinal cords possibly due to higher astrocytes and immature OLformation (skewed cell differentiation) after single/ fractionated dose of IR.

Prior to measurement of APE1, neuro-inflammation and changes in OPC in spinal cords, we used common test of memory (novel object recognition test (NORT) [30, 31]) to assess its relationship in conjunction with neuro-inflammation, alterations in APE1 and changes in white matter, in response to IR.

Thus, we hypothesize that altered BER caused by fractionated, low-dose and/or HZE radiation skews spinal cord OPC cell differentiation, resulting in inflammation and cognitive defects.

## Materials & Methods

### Ethics Statement

All exposures to radiation were done under i.p. anesthesia of xylazine/ ketamine mixture- 4.3–5 ml per rat (male, Wistar strain) for an average weight of 540–600 gms (80 mg/kg body weight of ketamine and 8 mg/ kg body weight of xylazine in PBS). Anesthetized rats’ spinal columns (thoracic portion) were exposed to low and high LET radiation (using single/ fractionated exposures of HZE- 300 MeV/n of ^28^Si/ 600 MeV/ n of ^56^Fe at 0.5Gy and 1Gy of 250 MeV Protons). After the fractionated exposure time points, we isolated spinal cords and brains from these rats, post-euthanasia at time intervals of 1.5, 3, 6 and 9 months post exposure. These tissues were fresh frozen and cryosectioned to stain for cell marker and TSPO (microglial) proteins. The method(s) of euthanasia was by inhalation of CO_2_, 100% CO_2_ at a rate of 20% air replacement per min. The indicators for euthanasia were no heartbeat.

These are consistent with the recommendations of the AVMA Guidelines on Euthanasia and the Center of Cancer Systems Biology/ GeneSys Research Institute’s Institutional Animal Care and Use Committee (IACUC) approved protocol. Anesthetized animals recover in about an hour and show no signs of distress from either X-rays or protons (1 Gy), after 5–9 months of single fraction exposure. We haven’t seen any visible distress of handling, transport or anesthesia complications in these animals. But with fractionated dose exposure, we had 1–2 rats (per each ion) exposed to protons and Si/ Fe come down with tumors, brain hemorrhaging and spinal column effects seen as bent spinal columns after 4–5 months post exposure, which were monitored regularly. These animals are monitored 2–3 times per week and when we saw signs of discomfort and debilitation like abnormal growth, curved spine, animal sickly and not feeding, that animal is sacrificed promptly.

### Irradiation set up for X-rays, protons and HZE ions exposure of spinal cords for rats

5 10–12 month old rats were used per fraction after anesthesia with Xylazine (8mg/ Kg body weight) / Ketamine (80 mg/ Kg body weight) i.p. 30 min. prior to their alignment with either lead collimator with a slit of 4 cm X 0.5 cm for X-rays in medical department, BNL or slit of similar dimensions made using tungsten blocks available at NSRL, BNL for exposure to HZE/ Protons. At NSRL, we used the Loma Linda holder to expose rats. Rats were exposed to single or fractionated dose of 1 Gy of 250 MeV protons (F1-1 Gy, F2- 0.5 Gy given in two fractions over 3 days, F3- 0.33 Gy per fraction given over 5 days) or 0.5 Gy of 300 MeV/n ^28^Si/ 600 MeV/ n ^56^Fe (F1-0.5 Gy, F2- 0.25 Gy given over 3 days, F3- 0.17 Gy given over 5 days with a day of no dose between fractions).

### Spinal cord section data

Spinal cords from rats exposed to 1Gy of X-rays/ 250 MeV protons/ 0.5 Gy of 300 MeV/n ^28^Si were isolated after 1.5–9 months post exposure, as per method detailed by Shihabuddin *et al*. [32] and the cords were frozen on dry ice. Different cell markers (A2B5-progenitor, NG2- Immature OL and GFAP-astrocytes) were measured along with APE1 in their OPC, to understand if there was a latent up-regulation of this BER protein, like that found by Kovalchuk *et al*, who found APE1 elevated for more than 4 weeks after exposure to low dose of 28 cGy per day for 27 days[25], and what was the consequence in terms of their OPC differentiation over time.

### Hematoxylin, APE1 and cell differentiation markers staining in spinal cord cryo sections

According to manufacturers’ instructions cryo-sections of the spinal cord (12 microns thick) were de-waxed using histo-clear (National Diagnostics, HS-200). The sections were rehydrated and the slides were washed with double distilled water before staining with Harri’s Hematoxylin (National Diagnostics HS-400) according to manufacturers’ instructions. The slides were rinsed with water and differentiated with 70% ethanol containing 0.1% HCl. The slides were again rinsed with water and stained with 1% aqueous Eosin with 0.1% acetic acid. The sections were dehydrated in 50, 95 and 100% ethanol followed by clearing with Histo–clear. The coverslip were mounted with Histomount (National Diagnostics HS-103). Duplicate sections were blocked in blocking solution of 1X PBS with 5% goat serum and 0.1% Tween 20 for an hour at 37°C. After two washes with wash buffer containing 0.1% BSA and 0.1% Tween 20 in 1XPBS, primary antibodies for APE1, A2B5, NG2 and GFAP (astrocyte marker) diluted at 1: 100–200 fold were added to the sections fixed on slides for an hour at 4°C and then washed twice with wash buffer and probed with appropriate Alexa Fluor antibodies to visualize the cell markers and APE1 changes 1.5–9 months post exposure. Antibody specificities for cellular (OPC, OL and Astrocytes) and protein (APE1,) markers were confirmed with appropriate positive and negative controls. GBM cell line (U251, overexpressing APE1 [33] to 10^7^-10^8^ molecules of APE1/ cell were used as positive control for APE1 (S1 Fig), while A2B5, NG2 and GFAP were confirmed by using negative controls of primary and secondary incubation without each other. Slides were imaged on Zeiss Axiovert Imaging system (Carl Zeiss, Germany) using 10- 40X or 63X objectives and appropriate filter sets (based on the excitation and emission wavelength of the fluorescent probes used) to detect each marker, keeping in mind that there is no spectral overlaps between the excitation and emission wavelengths of the probes. 2D images were acquired and stored on the microscope until analysis.

### APE1 Endo activity

APE1 endo activity was measured using a 51-mer duplex oligonucleotide substrate with a single abasic analog site (tetrahydrofuran) in one strand 3’ labeled with a tetramethylrhodamine fluorescent tag in the strand containing tetrahydrofuran (F) abasic analog. The assay buffer (50 mM Tris-HCl, pH 7.8, 100 mM NaCl, 10 mM MgCl_2_, 1 mM dithiothreitol) was described previously [34], and the incubation was at 37°C for 15 min. Following electrophoresis and imaging, samples with incision ≤50% were used to estimate the endonuclease activity.

### NORT

We measured its memory function by NORT. Memory function was assessed in control (n = 30), Protons-irradiated (Protons-irr) (n = 300), X-rays-irradiated (X-rays-irr) (n = 30) and Si/ Fe irradiated rats using published methodology[35] [31]. Briefly, on day1, habituation was performed by placing each rat in separate testing cage (a plastic box of 72 × 47 × 34 cm) for 1h. On the following day (for training), rats were placed in the same cage with two identical objects. The cumulative time spent by the rat exploring the two objects was recorded during a 5-min interval. A total of 4h later, the animals were reintroduced into the same cage for a 5-min test, where one of the two identical objects was replaced by a novel object. To offset location bias, the novel object was placed at the location of the old object, which the rat spent less time exploring (less than 45%) during the training phase. The time (out of the 3-min total) spent exploring each of the objects was recorded. The outcome measure was ratio of percentage of time spent exploring the novel object versus the familiar object during the testing phase, whereby normal healthy rodents are expected to spend relatively more time exploring a novel object than a familiar (i.e., ‘memorized’) object.

### Quantification of neuro-inflammation in rat brains from middle aged rats whose thoracic spinal cords were only exposed to 1 Gy of X-rays or 250 MeV Protons

As we were expecting that radiation will induce higher neuro-inflammation, it became important to measure radiation induced neuro-inflammation not only in rat spinal cords, but also in the rat brains from these rats.

### In Vitro [^3^H] Quantitative Autoradiography of translocator protein (TSPO), a microglial marker in rat brain sections

Following exposure and behavioral testing by NORT, rats were decapitated and brains were quickly removed, rinsed with 0.9% saline, and frozen in powdered dry ice. Long-term storage of brains was at −70°C. Frozen brains were sectioned in a cryostat (Jencons, OTF 5000) at −15°C in the coronal plane. Consecutive series of coronal brain sections (20 μm) were collected at 200 μm intervals from frontal cortex (∼3.7 mm from Bregma) to the cerebellum, covering the whole forebrain. On the day of the assay, sections were removed from –70°C and allowed to reach room temperature. TSPO autoradiography was performed with [^3^H] PK11195 (specific activity 83.5 Ci/mmol, PerkinElmer Life Sciences). PK11195 is used in positron emission tomography (PET) studies for imaging brain inflammation *in vivo* as it binds to the peripheral-type benzodiazepine receptor (PBR) expressed by reactive glia and macrophages, as previously described[36, 37]. Sections were first pre-incubated in 50mM Tris-HCl (pH 7.4) for 15 min at room temperature, followed by 30 min incubation (room temperature) with the radioactive ligand. Total binding was determined with 1nM ^3^[H] PK11195. Non-specific binding is determined on consecutive sections in the presence of excess (20μM) unlabeled PK11195. After completing autoradiography, sections were dried on a slide warmer at 60°C and loaded on KODAK BioMax MR films along with tritium micro scales for three weeks. After three weeks the developed films and digital autoradiograms were obtained by scanning the films on Umax 2100 scanner at 1600 dpi resolution. Quantitative image analysis was performed using ImageJ (NIH) software and manual drawings of regions of interest (ROI) on the accumbens (Acb), anterior Cingulate (Acg), cornu ammonis 1(CA1), cornu ammonis 3 (CA3) and dentate gyrus (dg), frontal cortex (Fr), Occipital cortex (Occ), Substantia Nigra (SN), Striatum (STR), and Ventral Hypothalamus (VHP) regions. ROIs were measured bilaterally, 3–6 measurements/ROI, and a mean value calculated for each animal/side. Nonspecific binding was subtracted from total binding to generate specific binding values translated to nCi/mg via a standard curve generated from the tritium standards. Autoradiography results were analyzed using Statview software. The groups (controls, X-rays and protons irradiated) were compared by two way ANOVA (treatment and region) followed by posthoc comparisons, using Fisher’s PLSD test when indicated. P<0.05 was considered significant.

## Results

**Fig 1:**
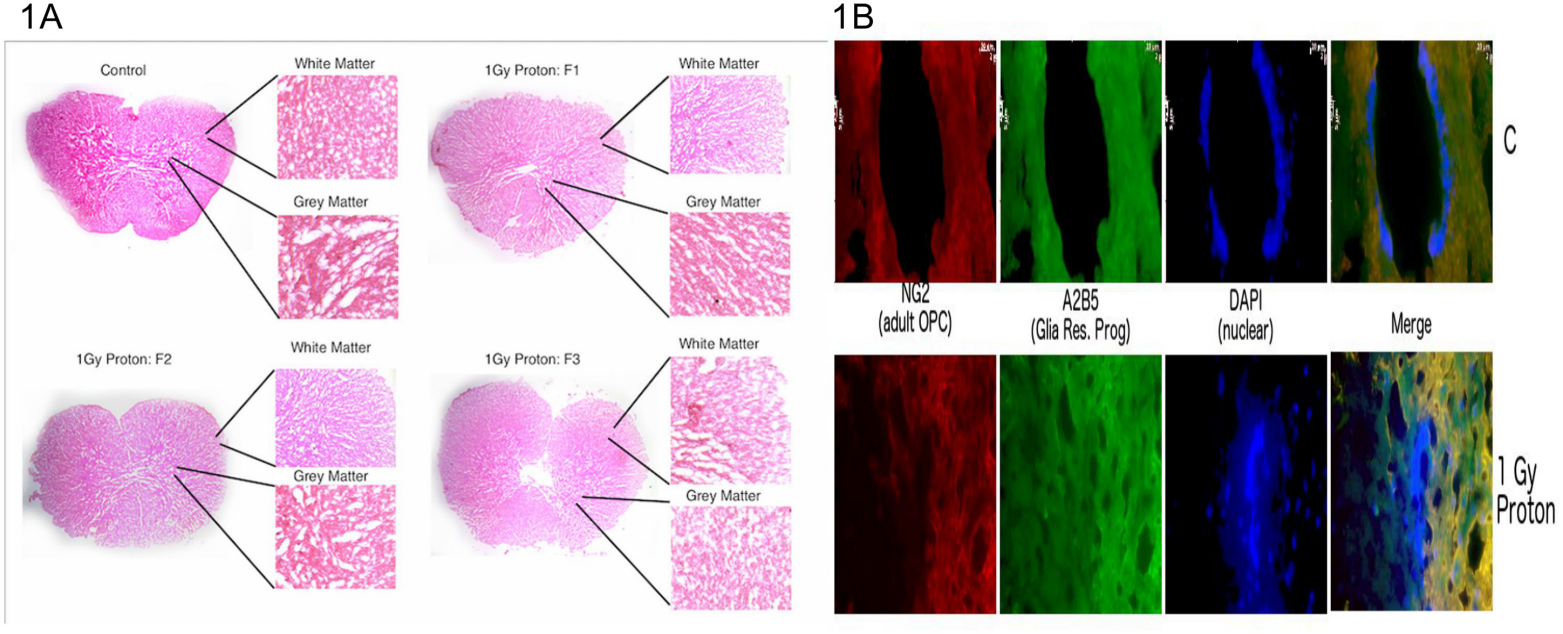
(A) H & E staining of spinal cord cryosections from protons exposed rats: 12 month old rats were exposed to 1 Gy of 250 MeV protons given as single (top right panel), fractionated [(2 fractions (lower left panel) and 3 fractions (lower right panel)] dose, their spinal cords were isolated 3 months post exposure, cryo preserved, thoracic T5 region sectioned into 12 μm sections, stained with H & E and imaged at 40X by fluorescent microscope available at Medical dept., BNL. Destruction of central canal, grey and white matter and increased proliferation of ependymal cells at central canal observed. (B) Cell markers staining of spinal cord cryosections from protons exposed rats: Duplicate T5 sections were stained with anti-NG2, A2B5 antibodies and DAPI and then imaged at Medical dept., BNL fluorescent microscope at 40X magnification in area of central canal. Destruction of central canal, increased proliferation of ependymal cells at central canal and lower NG2 staining observed.

Increased destruction of grey/ white matter, increase in proliferation of ependymal cells at the central canal and altered architecture of the thoracic spinal cord, 3 months post exposure to 1Gy of 250 MeV protons: Our present studies show that at latent time of 3 months post protons exposure, there is destruction of both grey and white matter of T7 thoracic section of middle aged rats’ spinal cord. H & E staining has been routinely used to study changes in spinal cord architecture after spinal cord injury (SCI) [38, 39]. As shown in Fig 1A, with fractionation, we find increasingly sparse white matter and grey matter along with increased distortion of the central canal. As shown in Fig 1B, protons also induced higher proliferation of ependymal cells of central canal, as measured by increase in nuclear staining indicative of inflammatory response with damaged central canal (as shown previously by Johansson *et al*.[40, 41]). Normally, cell division at the ependymal layer at central canal is rare [13]. When these sections were also stained with A2B5 (red), NG2 (green), we found the unexposed spinal cord section stain with both the cell markers, as seen with the good co-localization staining (orange), whereas the protons exposed spinal cord section showed decreased immature OL staining (much lower red/ orange stain indicating lower co-localization due to lower NG2 staining), indicative of decrease in OL progenitors over 3 months post protons exposure.

### Fractionation of ^28^Si causes decrease in progenitor cells and immature OL, with decrease in APE1 induction

**Fig 2:**
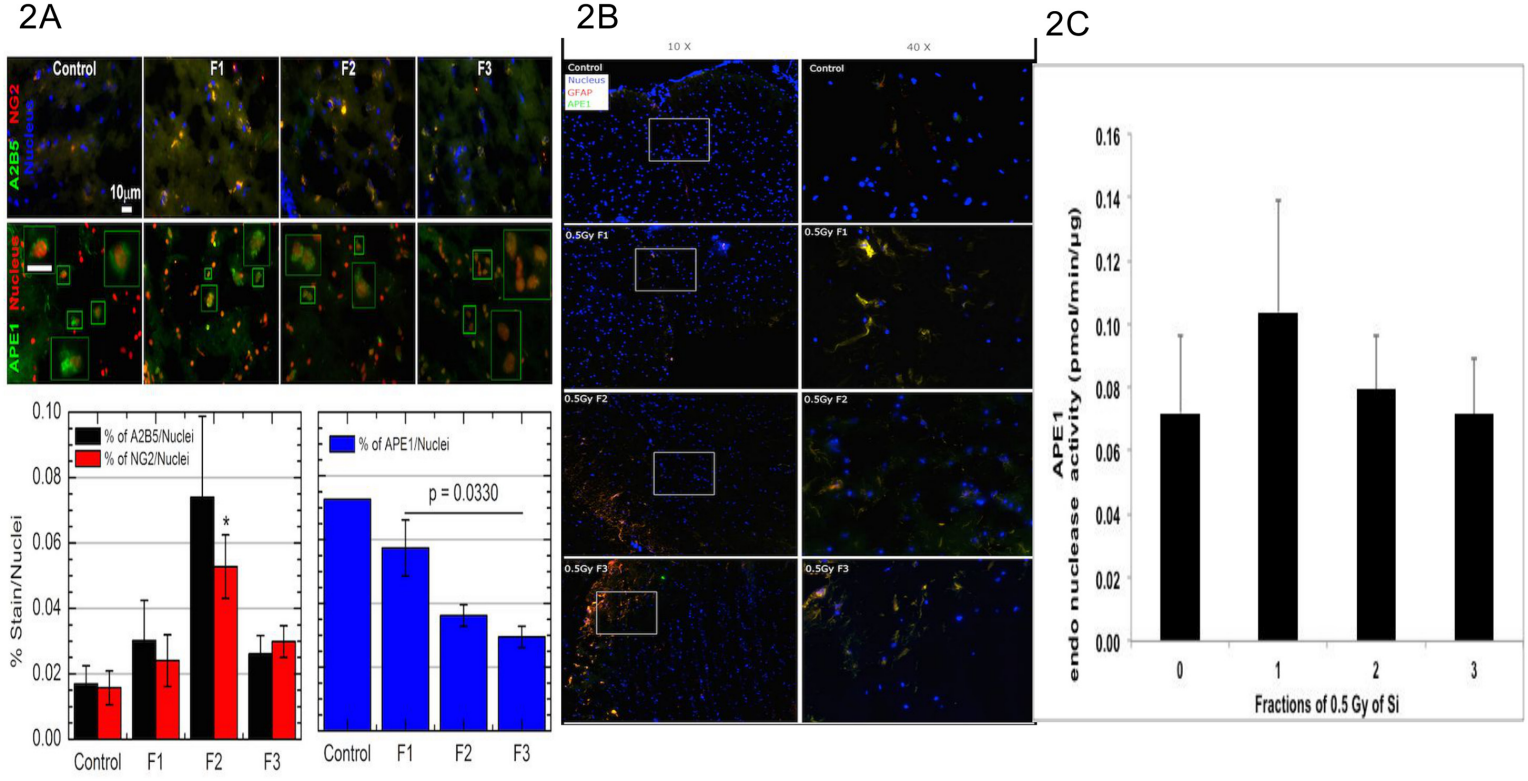
Cell markers staining of spinal cord cryosections from ^28^Si exposed rats. (A) Left image: Spinal cords from rats exposed to 0.5 Gy of 300 MeV/n ^28^Si,were isolated after 6 months post exposure. 8 μm thick cryosections from T7 (dorso cortico spinal (dcs) ventral column white matter section staining at 40X magnification is shown here) stained for APE1 and cell markers (A2B5, NG2), and imaged on Zeiss Axiovert Imaging system as described in Materials and Methods. Right image: Quantification of staining shown in left image. Total % of positive A2B5, NG2 and APE1 stains were measured and divided by the number of nuclei present in each field of view (n = 1 for one view). We averaged trend of staining per field of view and then averaged all the field of views. For those images we had n = 4 for all conditions except F1 where we had n = 3. The * represents p < 0.05 compared to the control. Other than that there was no significance between the conditions (although a trend is there). The scoring was obtained as an average of two independent investigators in a blinded fashion to nullify any bias which arise from just one investigator’s analysis. (B) Duplicate ventral column white matter section from Fig 2 samples, fixed and stained for APE1 and GFAP, imaged on FM at 40X magnification is shown here. (C) APE1 endonuclease activity measured from T7 sections of ^28^Si exposed rats’ spinal cords, 6 months post exposure by specific endonuclease assay described in methods. Increase in progenitor cell, immature OL and astrocytes seen up to two fractions, where as APE1 decreased with fractionation.

**Fig 3:**
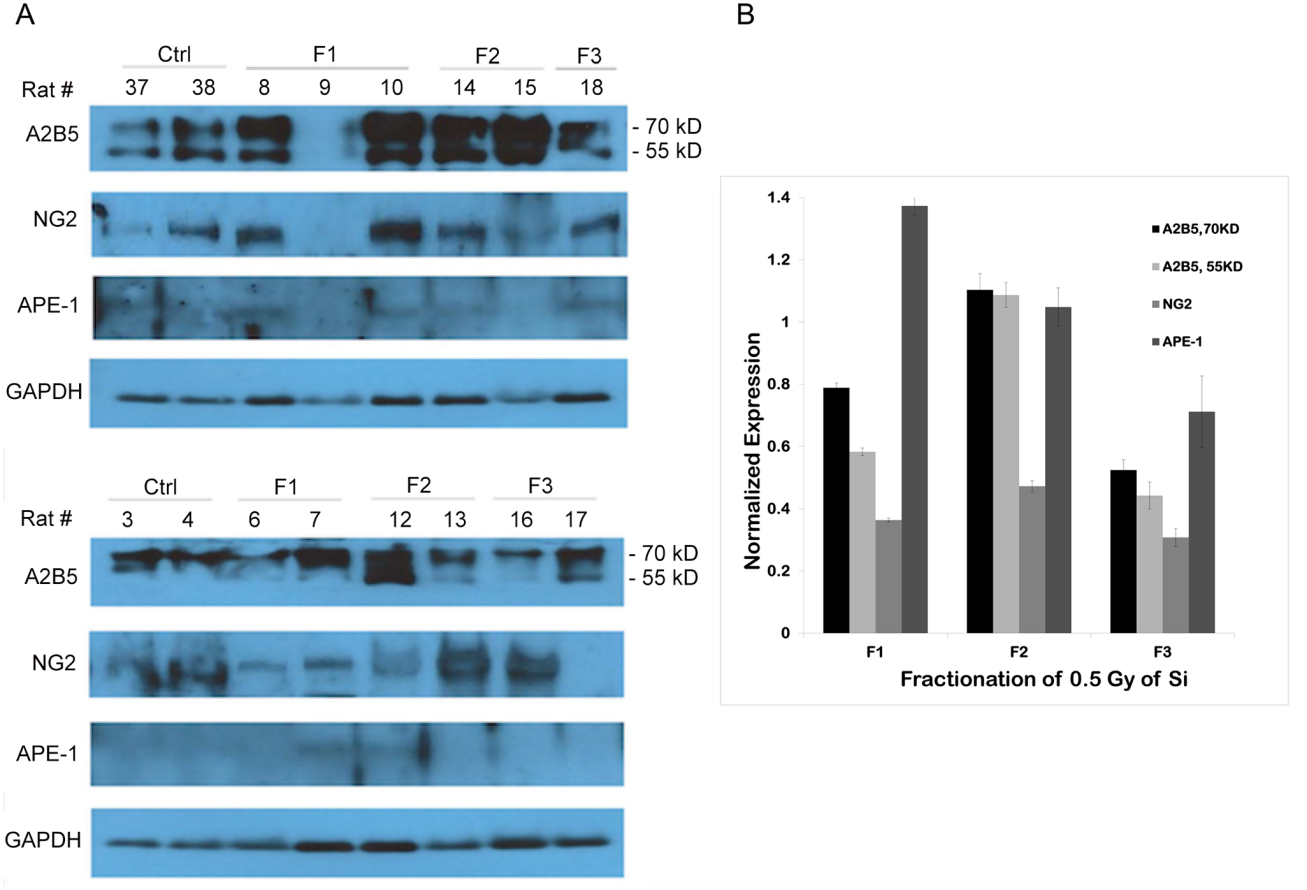
(A): left image. Western blot of thoracic (T5–T7) spinal cord extracts from 300 MeV/n ^28^Si exposed rats, harvested after 6 months post exposure measured for APE1 and cell markers (A2B5 and NG2). (B): right graph. Quantification of the 3A western blot with GAPDH control. Cell markers increased up to two fractions then decreased for third fraction, where as APE1 after a single dose induction, decreased with fractionation.

The effects of single/ fractionated doses of HZE on OPC cell differentiation, and APE1 were measured, revealing altered APE1 induction with most of it appearing outside the nucleus with single dose, which became increasingly nuclear with fractionation, and also showed initial increase in progenitor, immature OL (Fig 2A) and astrocytes (Fig 2B) staining which decreased after second fraction. Finally, when we assayed the T7 extracts for APE1 endonuclease activity, we saw a similar trend (Fig 2C) APE1 increases with single dose, but decreases with fractionation, although the total APE1 levels are too low to show statistically significant differences. Total protein extracts of the T7 sections were also measured for the same APE1, A2B5 and NG2 proteins by western blots (Fig 3), revealing a similar trend of increase in APE1 with single dose of Si that reduced when dose was fractionated. A2B5 and NG2 also showed similar trend to Fig 2B, thus corroborating the immunostaining data of sections.

### Protons and X-rays exposure at rat spinal cord results in very significant latent deficits in cognitive (memory) function

**Fig 4:**
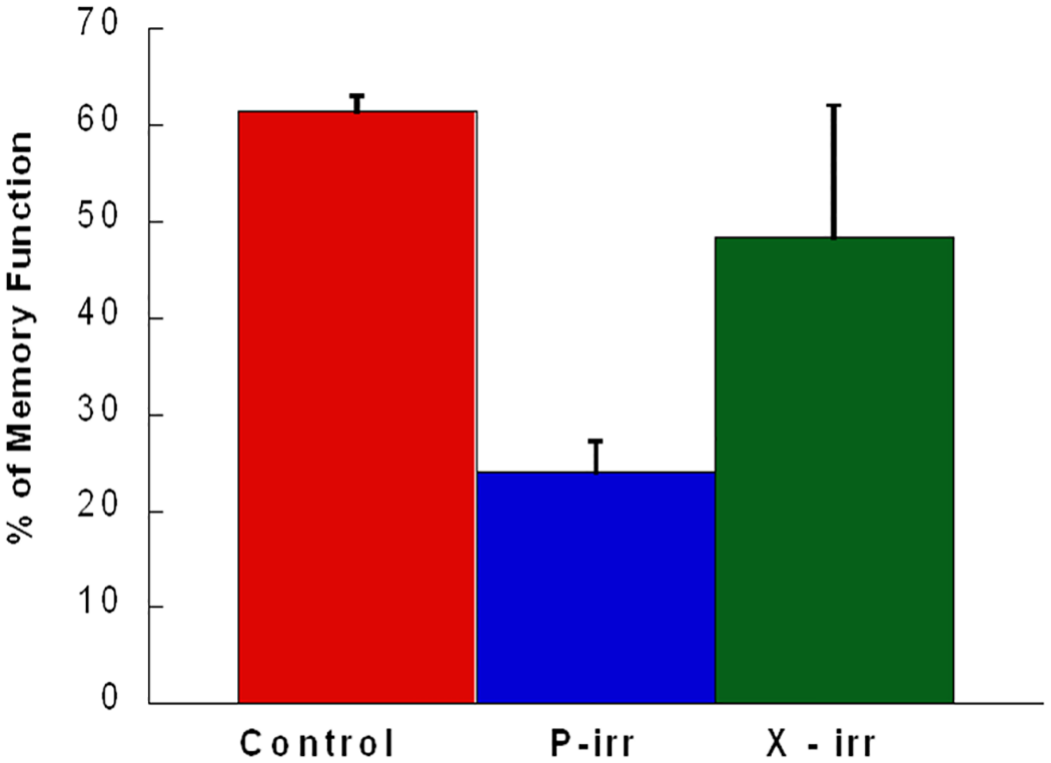
Novel Object Recognition Testing for low LET radiation exposed rats: Cognition testing for control, protons-irradiated (1 Gy of 250 MeV protons) (P-irr) and X-rays irradiated (1Gy X-rays) (x-irr) rats were performed at 9 months post exposure. Bars represent means and standard errors of the percentage of time spent by rats investigating a new object in the presence of a familiar one during a 3 min testing period. Memory function percentage revealed significant effect of exposure (p<0.05).

Animals were tested in the object recognition task at nine months after protons and X-rays exposure. Proton- irradiated animals showed higher deficit in this task, with lower preference for the novel object of 23.96± 1.68% as compared to X-rays irradiated animals which showed 48.32±13.69% (Fig 4), which was not significantly different from the percentage of time spent at the same location during the familiarization session. Controls performed consistently at all time points, showing a similar preference for the novel object upon repeated testing with new object pairs (Fig 4). Two-way ANOVA showed a significant difference between the Protons plus X-rays irradiated group and control group (p<0.05). Significantly cognitive deficit was observed more in Protons-irradiated (p<0.01) as compared to X-rays irrradiated (in trend 0.07) group.

### Neuro-inflammation

**Fig 5:**
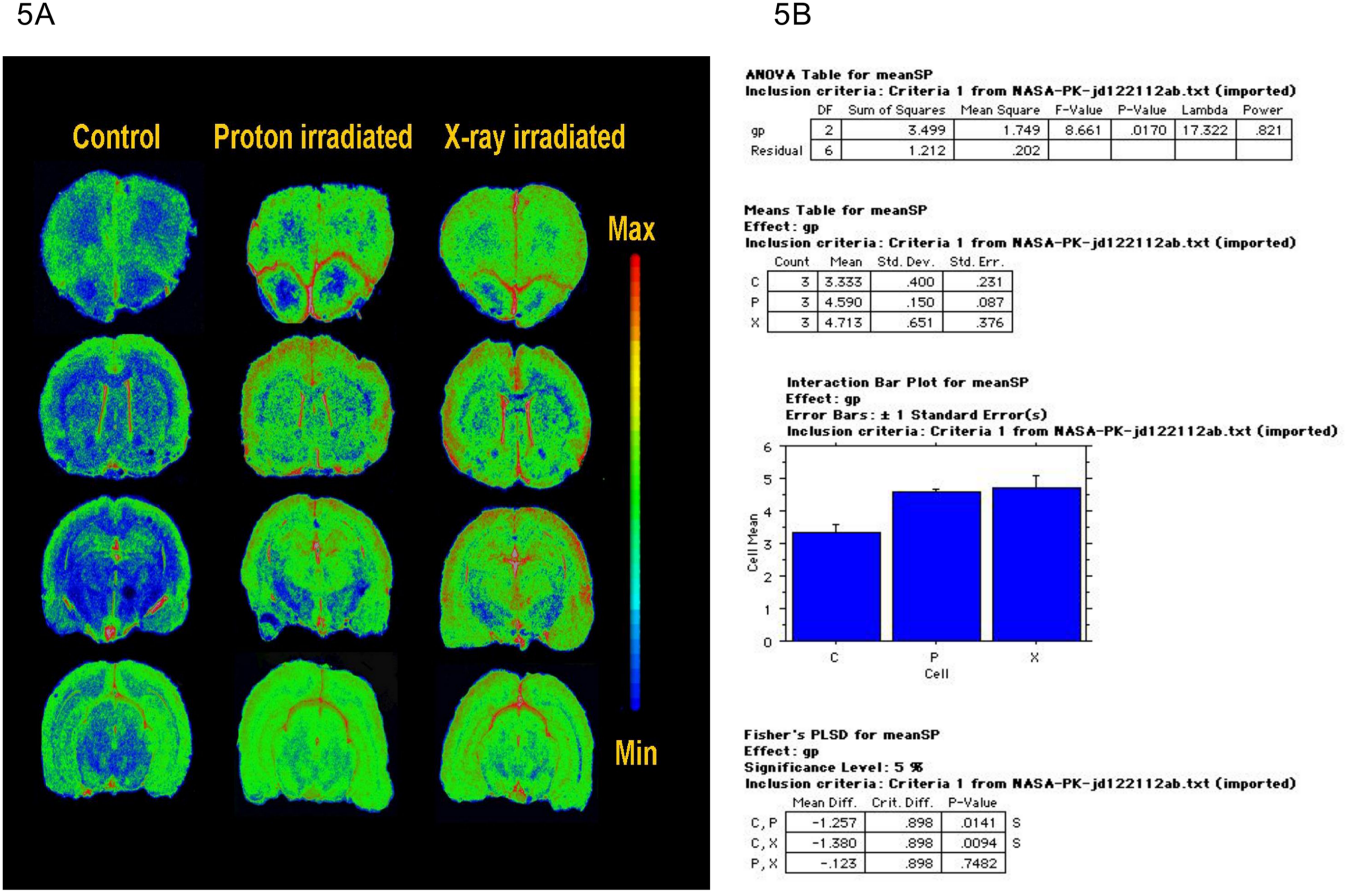
(A) Representative autoradiograms from control and irradiated (protons and X-rays) rats were pseudo-colored using the rainbow spectrum (scale on right). The neuro-inflammatory marker TSPO binding with [³H] PK11195 in rat brain sections is shown. A bilateral regional increase in inflammation is observed. (B) Statistical analysis is performed by 2-way ANOVA (treatment, time) with repeated measures (region). Alpha is preset at p<0.05. Regions of interest are grouped in four levels by distance from the impact (CHI), as follows: level 1 (mice, anterior striatal level, Bregma 1.7 to 0.02) contained sections in the direct path of the impact; level 2 (posterior striatal level, Bregma -0.22 to -0.82) contained regions directly posterior to the impact; and levels 3 and 4 (dorsal hippocampal level, bregma -1.2 to -2.06, ventral hippocampus/midbrain level, bregma –2.4 to– 3.6) contain regions relatively remote from the site of injury.

We were able to study these effects only 9 months post exposure, hence we have data from those rats brains, which indicate a clear increase in the microglial marker translocator protein (TSPO) binding in all brain regions including the hippocampus as shown in Fig 5A, with significant inflammation induced by X-rays and Protons with small number of rats (3 rats each for control, X-rays and Protons as 2 were sacrificed 3 months post exposure for spinal cord inflammation studies). Sections from control animals showed the known distribution of TSPO in the brain, with high density in ependymal cell layers associated with ventricles and relatively low and uniform levels in most brain regions (Table 1 and Fig 5B. ANOVA results showed a highly significant effect of treatment (F = 11.8, p<0.0001) as well as a significant effect of region (p = 0.0069) (Fig 4B). There was no treatment x region interaction (F = 0.19, p = 0.99) (Table 1).

**Table 1:** TSPO quantification in forebrain of X-rays and protons exposed (only at their thoracic spinal columns) rats: Results are mean +/- SE (Standard Error) of TSPO specific binding, expressed in nCi/mg in various brain regions of rat in control (n = 3), Proton irr = 3 and X-rays- irr = 3 rat brain regions after nine months of exposure to X-rays and protons to their thoracic spinal columns. Non-specific binding assessed in the presence of excess unlabeled PK11195 was subtracted from all measurements. Two ways ANOVA reveals highly significant effect between the group (p<0.0001) and of region (p<0.0069). Subsequent post hoc analyses were performed using Fisher's PLSD test.

Number	Region	Control	Protons-irradiated	X-rays-irradiated
		Mean+/-SE	Mean+/-SE	Mean+/-SE
1	Accumbens (Acb)	4.66 +/- 0.49	5.69 +/- 0.23	8.27 +/- 2.92
2	Anterior Cingulate (Acg)	5.44 +/- 0.22	7.06 +/- 0.39	10.28 +/- 3.82
3	Cornu ammonis (Ca A1)	2.71 +/- 0.27	4.02 +/- 0.10	5.97 +/- 2.68
4	Cornu ammonis (Ca A3)	2.35 +/- 0.18	3.35 +/- 0.16	6.69 +/- 3.28
5	Dentate gyrus (dg)	4.21 +/- 0.26	5.81 +/- 0.16	6.37 +/- 1.75
6	Frontal cortex (fr)	5.06 +/- 0.32	0.09 +/- 0.20	8.20 +/- 2.37
7	Occipital cortex (OCC)	3.33 +/- 0.31	4.60 +/- 0.90	4.71 +/- 0.38
8	Substantia nigra (SN)	1.95 +/- 0.30	3.31 +/- 0.27	5.17 +/- 1.68
9	Striatum (STR)	2.57 +/- 0.22	3.80 +/- 0.19	4.41 +/- 1.52
10	Ventral hypothalamus (VHPC)	2.57 +/- 0.24	3.73 +/- 0.40	5.72 +/- 2.27

### HZE and Protons exposure at rat spinal cord results in differential deficits in cognitive (memory) function at 6 months post exposure

**Fig 6:**
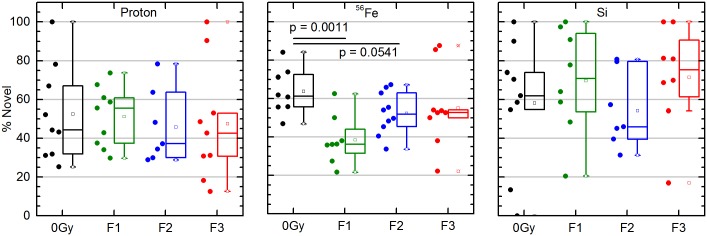
Cognition testing of control, protons (1 Gy of 250 MeV protons) (P-irr) and HZE exposed (0.5 Gy Fe/ Si) rats was performed at 6 months of post exposure. T test showed a significant difference between the Fe irr versus control group (p<0.002). 10 rats were analyzed per fraction, with 40 rats per ion (30 + 10 controls).

Animals were also tested in the object recognition task at six months after protons and HZE exposure. Fe irradiated rats showed most significant deficit with single dose and lower deficit with fractionation where as Protons as well as Si showed some trend towards more deficits with fractionation, although the data with Si NORT was most variable (Fig 6). Statistical analysis showed a significant difference only between the Fe-irradiated versus control group (p<0.0011 for single fraction and p<0.05 for two fractions). The single dose of Fe being most detrimental is expected with higher mass of Fe versus Si or Protons. These results are limited owing to the number of rats used (10/ fraction), as well as latent time points where with aged rats, we tend to lose 1–2 rats to premature sacrifice due to sickness.

## Discussion

Radiation research emphasizes understanding the mechanisms of radiation-induced DNA damage as one of the basic regulatory mechanisms governing the outcome. In this paper we have focused on identifying the changes in level of DNA repair protein APE1 induced by oxidative stress, due to HZE radiation in glial cells in comparison to low LET radiation from X-rays and protons. Ionizing radiation causes degeneration of myelin, the insulating sheaths of neuronal axons, leading to neurological impairment. Thus, astronauts exposed to protons and HZE radiation may risk adverse effects during their missions as well as latent health effects [42]. This paper is limited to some of the results obtained with low LET and high LET radiation. Oxidative DNA damage repair is known to be much less efficient in neurons and glia as compared to other cell types. Because the BER DNA repair protein APE1 is up-regulated in response to oxidative stress and is shown to cause inhibition of progenitor cells’ differentiation[26], it was important to determine if changes in APE1 affects the fate of OPC, which replace the damaged mature OL, causing a skewing of this differentiation, resulting in higher astrocytes, and subsequently a higher inflammatory response. Thus, our current studies focused on determining whether altered BER mechanism could result in altered OPC differentiation, causing demyelination, neuro-inflammation and cognitive changes in response to radiation injury, with an aim to determine a possible link between cognitive deficit, DNA repair and neuro-inflammation.

Our present results show some inflammation of rat spinal cord (increased ependymal cell proliferation) and increasingly sparse grey/ white matter architecture with fractionated dose of protons, indicating a possible causal link. There was also a increase in nuclear localization of APE1 in spinal cord OPC, with fractionation of ^28^Si, but with decrease in total APE1 protein (except the initial increase seen with single dose) without any significant change in its APE1 endo activity, indicative of single dose inducing APE1, and with HZE dose fraction likely due to more damage, the cells show lowered APE1. As the amount of endonuclease activity detected was so low (0.065 pmoles in control), one cannot conclude if there were changes in its activity as is shown in the APE1 protein content. Further, fractionation resulted in increase in progenitor, immature OL and astrocytes cell types and caused a significant inflammatory response even in those tissues which were not irradiated (increased microglial marker in the brain, Fig 5A and 5B), possibly via APE1 induced activation of STAT3 resulting in increased in pro-inflammatory cytokines such as IL-6 [43].

These findings align with previous reports on irradiation induced neuro-inflammation and the ability of neuro-inflammation to spread from the site of injury to relatively remote regions of the central nervous system [36, 44].

Recently, we have also measured these rats heart blood and tissue extracts for changes in inflammatory cytokines, TNF-α and NFκB and found an increase in NFκB and changes in serum TNF-α (S1 Fig), indicative again of increased inflammatory response.

The increased survival of neonatal and young O-2A progenitor cells observed after fractionation with X-rays or fission neutrons can be attributed to repopulation rather than repair[45]. IR induces dysfunction in neural-precursor cells by altering the microenvironment that regulates their fate and proliferative capacity. In fact, the irradiated progenitor cells generated more immature OL that failed to differentiate further into mature OL *in vivo*[46]. We show a similar trend in our studies with increase in OPC and immature OL.

As previous studies with 250 MeV protons in 2 month old rats [47] hadn’t elicited any deficit in behavior, we wanted to determine if an effect could be elicited in older rats (10 months at exposure) at comparable age (45–55 years) to astronauts and we found cognitive defects with protons being more effective at same dose than X-rays, but at latent time point of 9 months post exposure. When these rats were measured for cognitive deficit at 6 months (Fig 6), we didn’t find a significant deficit with protons as seen 9 months post exposure (Fig 4), despite a trend for it that increased with dose fractionation. As expected ^56^Fe was most detrimental when given as a single dose and deficit declined with dose fractionation. ^28^Si effects seemed to show trend towards higher deficit with fractionation, although without a level of significance to this trend. We may have seen them achieved if we could have kept these animals for 9 months post exposure, but these rats show more sickness (tumors and weight loss) with time, so they needed to be sacrificed earlier. Age is another limitation as these studies were done in middle aged rats which become old when they are measured for NORT (10–12 month old rats which were measured for NORT 6–9 months post exposure), so one has to consider the effects of aging exacerbating the decrease in cognitive abilities.

Thus, for the first time we have established that fractionation of radiation causes higher degradation of grey/ white matter, inflammation and altered APE1 levels (and its localization indicating the HZE effect seems more nuclear than cytoplasmic), increased OPC, immature OL, and astrocytes in spinal cord and a very significant neuro-inflammation in brain, which likely leads to the observed significant cognitive defects.

## Supporting Information

S1 Fig Inflammatory molecules measurement from Rats’ heart tissue (NFκB) and sera (TNF-α) harvested 9 months post exposure to 0.5 Gy of single/ fractionated dose of 300 MeV/ n ^28^Si, at their thoracic spinal columns. NFκB was measured from heart tissue by the ChIP assay and TNF-α was measured in rat heart sera using bioactive assay.
(TIF)
