# Supplementary figures and images for: Fractionated Radiation Exposure of Rat Spinal Cords Leads to Latent Neuro-Inflammation in Brain, Cognitive Deficits, and Alterations in Apurinic Endonuclease 1
Source: PLoS One. 2015 Jul 24;10(7):e0133016. doi: 10.1371/journal.pone.0133016 (PMC4514622; doi:10.1371/journal.pone.0133016)

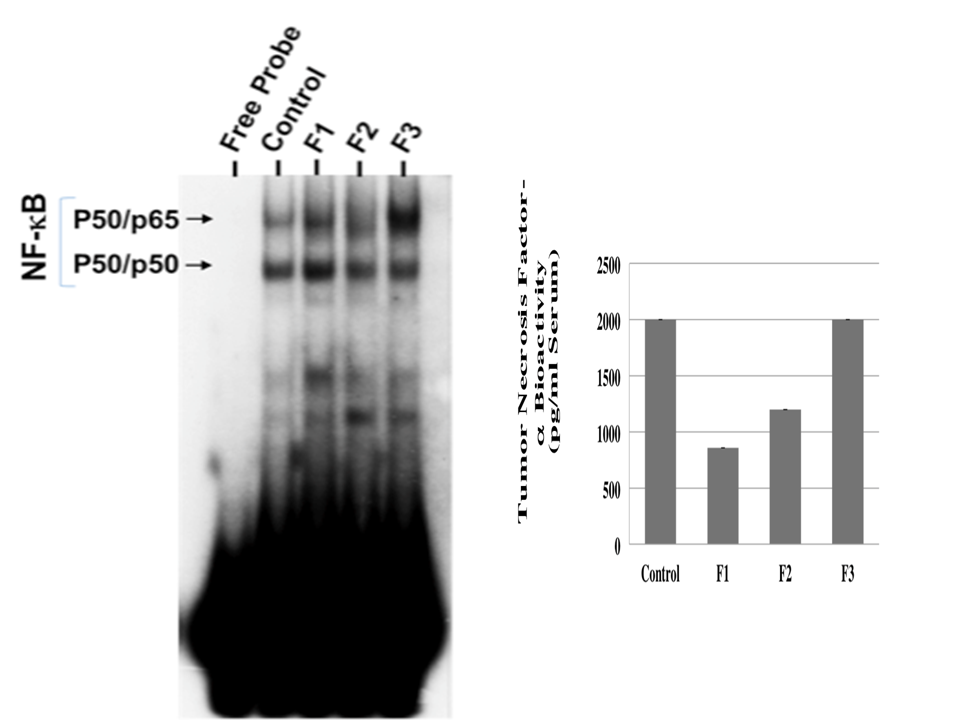

Supplement: S1 Fig — NFκB was measured from heart tissue by the ChIP assay and TNF-α was measured in rat heart sera using bioactive assay. (TIF) [file pone.0133016.s001.tif]
